# Synthetic Whole-Cell Biodevices for Targeted Degradation of Antibiotics

**DOI:** 10.1038/s41598-018-21350-9

**Published:** 2018-02-13

**Authors:** Peng-Fei Xia, Qian Li, Lin-Rui Tan, Miao-Miao Liu, Yong-Su Jin, Shu-Guang Wang

**Affiliations:** 1 School of Environmental Science and Engineering, Shandong University, 27 Shanda Nanlu, Jinan, 250100 P.R. China; 2 Department of Civil and Environmental Engineering, University of Illinois at Urbana-Champaign, 205 North Mathews Ave, Urbana, IL 61801 United States; 3 Carl R. Woese Institute for Genomic Biology, University of Illinois at Urbana-Champaign, 1206 West Gregory Drive, Urbana, IL 61801 United States; 4 Department of Food Science and Human Nutrition, University of Illinois at Urbana-Champaign, 905k South Goodwin Avenue, Urbana, IL 61801 United States

## Abstract

Synthetic biology enables infinite possibilities in biotechnology via employing genetic modules. However, not many researches have explored the potentials of synthetic biology in environmental bioprocesses. In this study, we introduced a genetic module harboring the codon-optimized tetracycline degrading gene, *tetX.co*, into the model host, *Escherichia coli*, and generated a prototypal whole-cell biodevice for the degradation of a target antibiotic. Our results suggested that *E. coli* with the *tetX.co-*module driven by either the P_J23119_ or P_BAD_ promoters conferred resistance up to 50 μg/mL of tetracycline and degrades over 95% of tetracycline within 24 h. The detoxification ability of *tetX* was further verified in conditioned media by typical *E. coli* K-12 and B strains as well as *Shewanella oneidensis*. Our strategy demonstrated the feasibility of introducing genetic modules into model hosts to enable environmental functions, and this work will inspire more environmental innovations through synthetic biological devices.

## Introduction

Advances in DNA reading and writing techniques, as well as developments in genetic circuits, have enabled synthetic biological designs in microorganisms for precise purposes^1–3^. Due to these achievements, metabolic engineering and synthetic biology have made tremendous progress in heterologous pathway integration^4,5^, genetic toolkit developments^6^, and physiological function discoveries^7,8^. However, this progress has mainly focused on the fields of biofuel and value-added chemical production, medicine discovery, and genetic diagnoses and therapies. Environmental biotechnology is one of the most important sectors of applied microbiology, but to our knowledge, not many examples exist of synthetic biology approaches applied for environmental biotechnology purposes.

Environmental biotechnology has been extensively utilized in water and wastewater treatment, soil remediation, and sludge disposal^9–11^. Functional microbial consortia are the main participants in almost all bioprocesses, especially in wastewater treatment, such as activated sludge^12^, aerobic or anaerobic granules^13^, and biological electrosystems^14,15^. Despite of these mixed cultures, microbial strains with novel physiological functions have been defined, such as *Nitrosomonas* sp., *Nitrosococcus* sp., *Nitrospira* sp., and *Dehalococcoides mccartyi*^16–20^. The direct utilization of pure cultures with single strains of microbes harboring unique functions is, nevertheless, a significant challenge.

Most widely used microbial strains in environmental biotechnology have been identified because of their distinct and innate properties instead of being engineered from model strains with integrated genetic modules. Compared with the fields where synthetic biology and metabolic engineering have prospered, similar scenarios can be developed in environmental biotechnology. Microorganisms with unique and desirable functions usually experience native bottlenecks like low growth rate, harsh cultivation conditions, and limited genetic information^16,20^, while model hosts, such as *Escherichia coli* and *Saccharomyces cerevisiae*, although amenable to genetic manipulation and easy to cultivate, lack desired functions^21^. Therefore, it would be a promising alternative if we transplant particular functions of environmental microorganisms into model hosts with well-established genetic information and toolkits.

In this endeavor, we designed and introduced a genetic module capable of degrading antibiotics into *E. coli*, to examine whether a model microbial host could be engineered to a whole-cell biodevice for environmental purposes. On behalf of environmental science, antibiotics are not only pollutants of particular concerns but also harbor antimicrobial effects, which severely impact the existing water and wastewater treatment bioprocesses^22–24^. As a proof of principle, we chose tetracycline, a typical antibiotic detected in nature and environmental facilities, as our target, and the *tetX* gene from *Bacteroides fragilis*^25,26^ was codon-optimized (denoted as *tetX.co*) and expressed in *E. coli* BW25113 strain (briefed as BW25113 hereafter). Unlike other tetracycline resistance genes used in molecular biology, *tetX* confers resistance against tetracycline via bioconversion and detoxification rather than blocking the antimicrobial machinery of tetracycline^26^. The resistance and degradation capability toward tetracycline plus the detoxification ability of *tetX* have been demonstrated and discussed.

## Results and Discussion

### Designing genetic modules for the degradation of tetracycline

**Figure 1:**
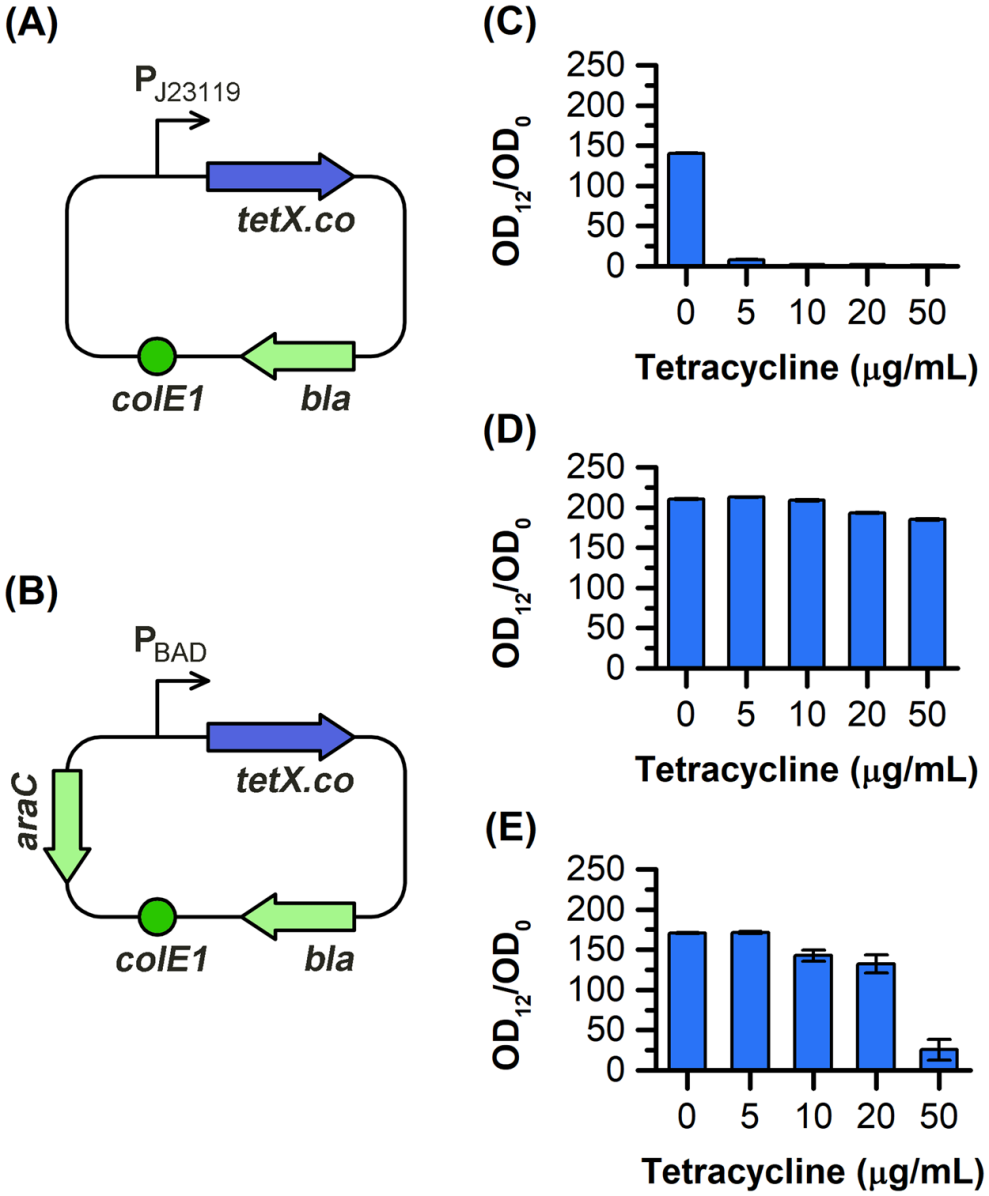
Design schemes of plasmids with *tetX.co* led by P_J23119_ pTc, (**A**) and P_BAD_ pTi, (**B**) promoters, and the resistance of BW25113 with empty plasmid (**C**), pTc (**D**), and pTi (**E**). The cells were first cultured overnight and inoculated into LB media with different concentrations of tetracycline, the resistance was represented by OD_12_/OD_0_. All experiments were conducted at least in duplicate and error bars denoted to ± SD.

To construct the genetic modules, the *tetX* gene from the transposon Tn4351 in *B. fragilis* was codon optimized for better expression in BW25113 and the resulting *tetX.co* was fused to the pBAD backbone plasmids led by either a constitutive (P_J23119_) or an inducible promoter (P_BAD_) with different strengths (Fig. 1A and B), resulting in pTc and pTi, respectively. The pTc (*tetX.co* led by P_J23119_) and pTi (*tetX.co* led by P_BAD_) were then transformed into BW25113. The transformants with the designed plasmids together with the control strain (BW25113 with empty plasmid) were tested whether they could survive in different concentrations of tetracycline.

The results showed that the control strain did not grow even in the presence of only 5 μg/mL of tetracycline in LB media (Fig. 1C). The BW25113 with pTc or pTi, as expected, could grow under various concentrations of tetracycline up to 50 μg/mL via a regular 1/100 ratio inoculation at the beginning of cultivation. For BW25113 harboring pTc, the cells reached up to 185.14 ± 1.27 OD_12_/OD_0_ in the presence of 50 μg/mL (Fig. 1D), indicating the successful functionality of *tetX.co* led by the constitutive promoter P_J23119_. The BW25113 harboring pTi also grew well in the presence of 50 μg/mL of tetracycline with the OD_12_/OD_0_ of 25.47 ± 12.69 (Fig. 1E). This was further verified at all tested levels of tetracycline, suggesting that *tetX.co* was functional and conferred the tetracycline resistance in *E. coli*. Meanwhile, we discovered that a pre-accumulation of TetX was necessary when higher levels of tetracycline were present in the media. When we first cultivated the BW25113 with pTi without the inducer, *L*-arabinose, the cells could not grow well when inoculated in fresh LB media with 20 and 50 μg/mL of tetracycline. However, the strain harboring pTc (*tetX.co* was expressed constantly) was able to grow in the same conditions, suggesting the requirement of pre-accumulated TetX for cells to survive in high levels of tetracycline. Then, we added *L-*arabinose during preculture of BW25113 harboring pTi, and identified the promising growth profile of this strain in the presence of 50 μg/mL tetracycline (Fig. 1E).

### *In vivo* conversion of tetracycline by *E. coli* with tetracycline-degrading modules

As reported, a functional TetX was able to convert the tetracycline into 11a-hydroxy-tetracycline through the hydroxylation of tetracycline in the presence of oxygen and NADPH. Although *in vitro* study has demonstrated the bioconversion pathway, whether the codon optimized TetX in model hosts could convert tetracycline as expected *in vivo* was still unknown, as heterologous enzyme may fold differently between *in vivo* and *in vitro*^27^. As such, the converted tetracycline by our biodevice was examined using spectrophotometry and MS analysis. The BW25113 harboring pTc or pTi was first cultured in LB media overnight without tetracycline, and, after washing twice with M9 media, the cells were inoculated into fresh M9 minimal media with initial OD_600_ 1.0 to avoid unexpected background noise from complex rich media. The tests were conducted at 220 rpm and 37 °C to ensure sufficient aeration, and the samples taken at different time intervals were filtered and analyzed by UV-Vis spectra and MS analysis.

**Figure 2:**
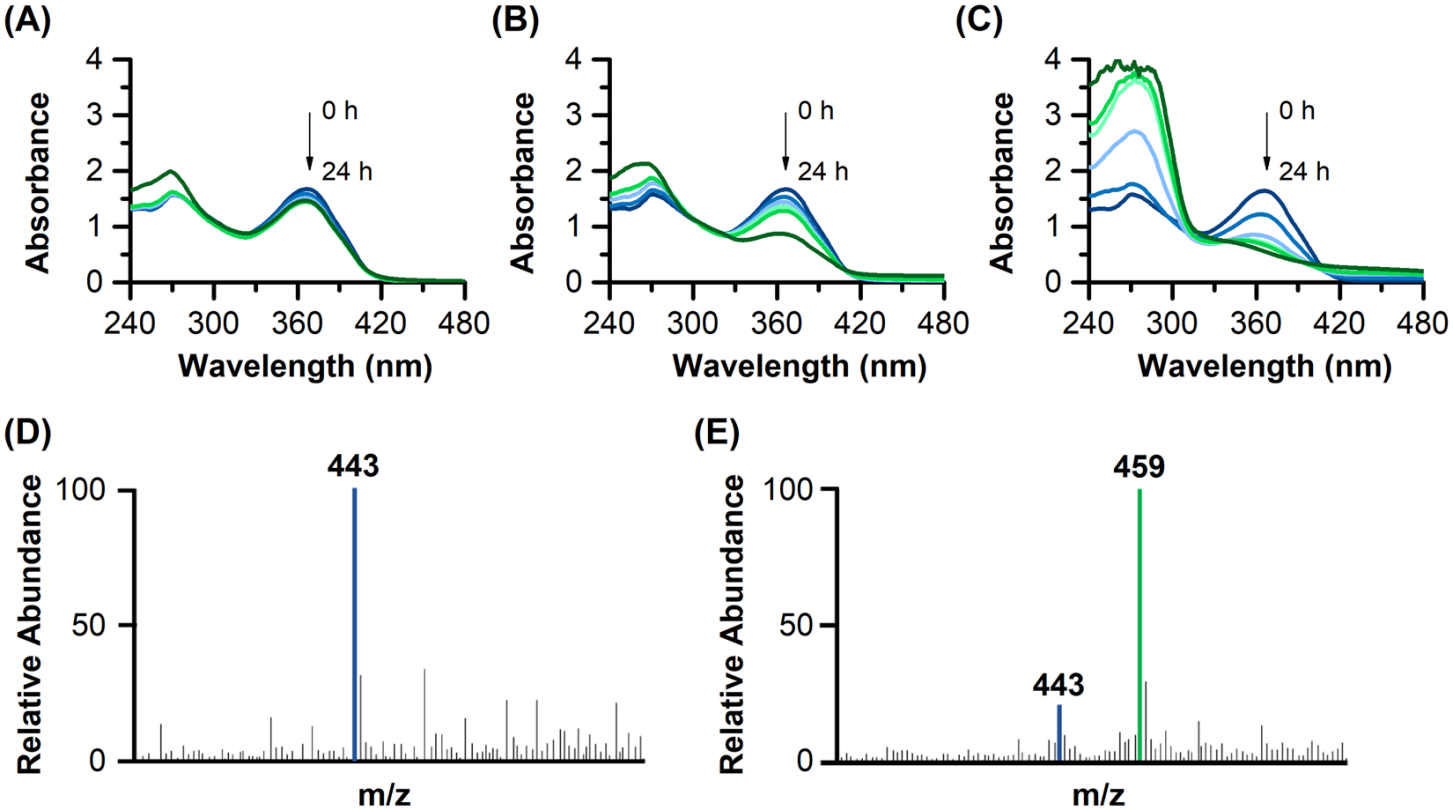
UV-Vis spectra of the filtered M9 media with 50 μg/mL of tetracycline cultured with BW25113 with empty plasmid (**A**), pTc (**B**) and pTi (**C**). The MS of the M9 media with 50 μg/mL of tetracycline (**D**) and those cultured with BW25113 with pTi for 24 h (**E**). The MS data were shown from 400 m/z to 500 m/z and represented by relative abundance.

The UV-Vis spectra of the supernatant from the control strain did not reveal significant differences at the wavelength of 365 nm in 24 h (Fig. 2). The slight decrease of absorbance might be attributed to the partial adsorption of tetracycline on cell surfaces rather than degradation, which might also occur at the beginning stage of our engineered strains (Fig. 2A). On the contrary, we clearly identified the decrease of absorbance at 365 nm for strains with pTc and pTi (Fig. 2B and C). For BW25113 with pTc, the absorbance at 365 nm decreased gradually from the first 12 h, and sharply reduced at 24 h, and an increase at 260 nm was also identified. These absorbance changes might result from the conversion of tetracycline and non-enzymatic breakdown of the converted chemicals^26^. For the strain with pTi, the absorbance at 365 nm quickly decreased even from the first sampling time (3 h), and at 24 h, almost no absorbance at 365 nm was observed, indicating a complete conversion of tetracycline. These results suggested the conversion of tetracycline, and the strong expression of *tetX.co*, which regulated by the P_BAD_ promoter, led to faster degradation under the same conditions. Then, the MS was employed to analyze the products in the culture of BW25113 with pTi. The peak at 443 m/z was clearly identified in M9 media with 50 μg/mL of tetracycline (Fig. 2D). After 24 h, the abundance of the peak at 443 m/z decreased, while a new peak at 459 m/z appeared, indicating the hydroxylation of tetracycline (Fig. 2E). Therefore, UV-Vis and MS data provided evidence that the BW25113 strains with the functional TetX efficiently degraded tetracycline through hydroxylation.

### Degradation kinetics of tetracycline by *E. coli* harboring the *tetX.co* module

**Figure 3:**
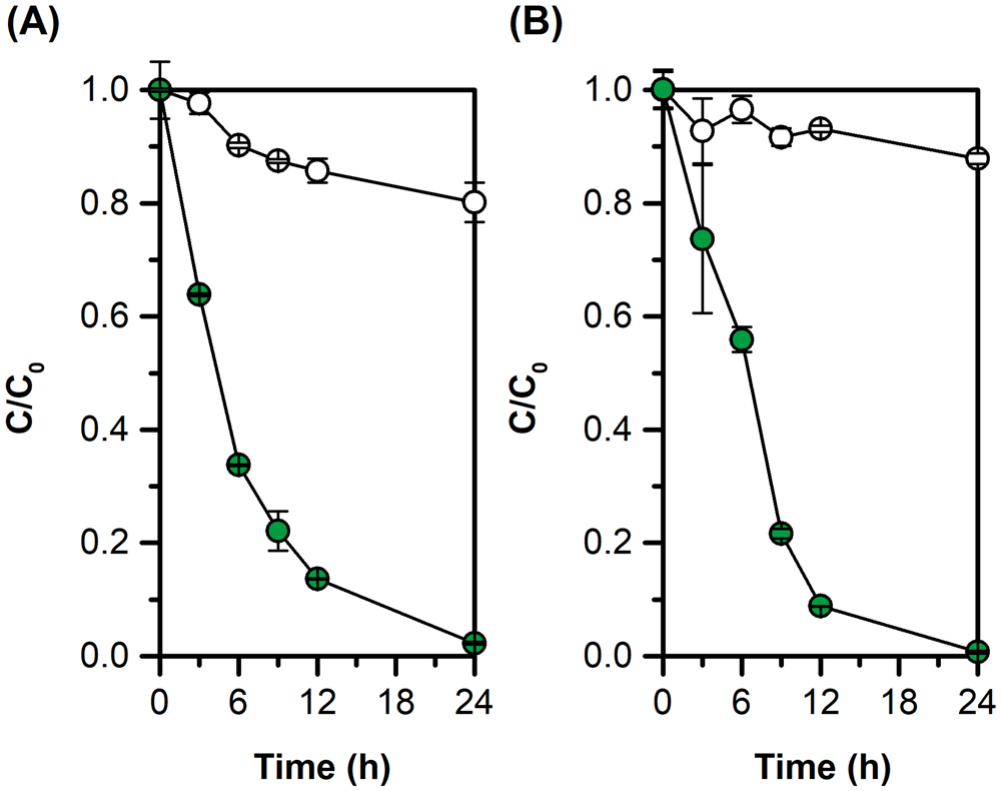
Degradation kinetics of tetracycline by BW25113 with pTi in M9 media (**A**) and LB (media). The testing strain was cultured overnight in LB with *L*-arabinose and inoculated in M9 and LB media with 50 μg/mL of tetracycline, respectively. The samples were taken at different time intervals and measured by HPLC. All experiments were conducted in duplicate and error bars show the ± SD.

The degradation kinetics of *E. coli* with pTi were further analyzed in minimal and rich media to determine the degradation capability of our engineered strain with *tetX.co* led by the strong promoter pBAD. The cells with pTi were first cultivated in LB media with *L-*arabinose overnight and then inoculated into M9 and LB media with initial OD 1.0 and 50 μg/mL of tetracycline. The samples were taken at different time intervals and tetracycline concentrations were measured by HPLC. As shown in Fig. 3A, BW25113 with pTi degraded tetracycline efficiently within 24 hours in M9 media. At 3 h, over 35% (C/C_0_ 0.638 ± 0.002) of the tetracycline was converted and at 24 h over 95% (C/C_0_ 0.023 ± 0.002) of tetracycline was degraded in M9 media. Meanwhile, only 20% (C/C_0_ 0.801 ± 0.035) of the total tetracycline was removed from the media for the control strain, which might result from the adsorption. Similar trends were found in the LB media with some slight differences, and the *E. coli* cells in LB media degraded the tetracycline more completely at 24 h (99%, C/C_0_ 0.007 ± 0.002).

### Detoxification analysis of degraded tetracycline

Although TetX cannot fully degrade tetracycline into small molecules, such as CO_2_ or ethanol, it addresses the first and the most important step of tetracycline degradation, detoxification. Our engineered *E. coli* with *tetX.co* destroys the antimicrobial functions of tetracycline and facilitate the subsequent degradation. Therefore, the detoxification effects of TetX were analyzed to test the utility of our synthetic biological strategy. To do so, we selected the typical *E. coli* K-12 MG1655, B strain BL21 as well as *Shewanella oneidensis*^28,29^, to evaluate whether the conditioned media (CM) of our engineered strain, the supernatant from the culture of the control and our engineered strain with 50 μg/mL of tetracycline, were detoxified.

**Figure 4:**
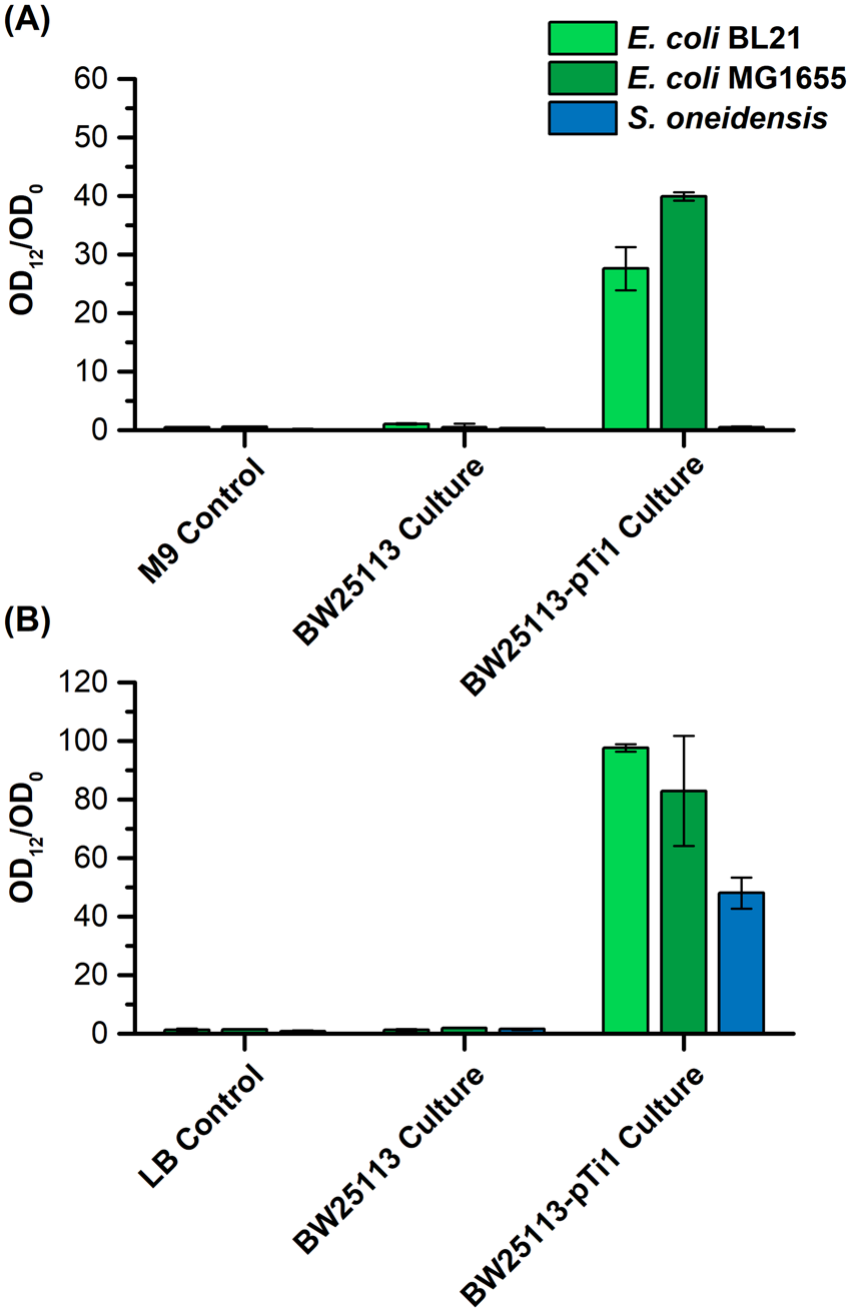
Detoxification tests of converted tetracycline using conditional media from either the minimal media (**A**) or rich media (**B**). The BW25113 with pTi were overnight cultured and inoculated in M9 and LB media with 50 μg/mL of tetracycline, respectively. After 24 h, the culture was centrifuged and filtered, then regarding as CM. *E. coli* MG1655, BL21 as well as *S. oneidensis* were inoculated into CM and the OD_12_/OD_0_ were recorded to evaluate the detoxification ability of TetX. All experiments were conducted in duplicate and error bars show the ± SD.

As shown in Fig. 4A and B, all three tested strains did not grow in pure media with 50 μg/mL of tetracycline within 12 h. Also, these strains were not able to survive in the CM of BW25113 with the empty plasmid, indicating that the control strain could not detoxify tetracycline as expected. We harvested the CM of our engineered strain after cultivating for 24 h in M9 (CM_M9_) and LB (CM_LB_) media, respectively. Both the *E. coli* MG1655 and BL21 grew well in the CM_M9_, indicating the tetracycline in the original M9 media has been detoxified, and the CM_M9_ allowed wild-type strains grow (Fig. 4A). As *S. oneidensis* could not grow in even the original M9 media (data not shown), the *S. oneidensis* did not grow in the CM_M9_. Then, we evaluated the effects of CM_LB_ of our engineered strain on all three tested strains. First, we identified the growth of *S. oneidensis* in CM_LB_, indicating that the converted tetracycline lost most of the antimicrobial effects on *S. oneidensis* after conversion by BW25113 with pTi. Moreover, we tested the CM_LB_ with the two *E. coli* strains. Glucose with the final concentration of 4 g/L was added into the CM_LB_ before inoculation to supply nutrients, and both strains grew well in the CM_LB_ of BW25113 with pTi, which in addition evidenced the promising detoxification capability of TetX in rich media. The slightly lower OD_12_/OD_0_ of *E. coli* BL21 and MG1655 might attribute to the depletion of substrates and the residue tetracycline (0.47 ± 0.15 μg/mL) in the CM.

### Bridging the synthetic biology and environmental biotechnology

Selection of proper microorganisms is the most important step in biotechnology^30,31^. Most applied bioprocesses for environmental purposes prefer mixed cultures because of the resistance and tolerance toward toxic chemicals, varied operating conditions and other detrimental shocks. However, even mixed culture cannot thrive in some harsh conditions, such as wastewater with high concentrations of antimicrobial chemicals. As reported, traditional biological treatment processes could only remove 11.6% to 85.4% of the total tetracycline^32^, and our previous work demonstrated that 83.6% of the tetracycline at an initial concentration of 10 μg/mL could be removed by enriched mixed culture^33^. Nevertheless, the activity and function of the mixed culture were severely inhibited by tetracycline at this level^33,34^. In pharmaceutical industry, the concentration of tetracycline could reach up to 19–32 μg/mL (mg/L), which was a grand challenge to current treatment procedures^35,36^.

Synthetic biology and metabolic engineering provide an alternative approach to overcome the limitations inherent in traditional strategies via introducing genetic modules into model microorganisms. This can be easily cultivated and manipulated at various levels. To prove this concept and propose a prototype to bridge synthetic biology and environmental bioprocess, we chose the typical antibiotic, tetracycline, as our environmental target, and the *tetX* gene was selected to construct our genetic modules. The efficient conversion of tetracycline via hydroxylation, the degradation kinetics, as well as the detoxification of tetracycline by TetX have been evaluated *in vivo*. According to our results, the designed whole-cell biodevice was able to grow in the presence of 50 μg/mL tetracycline and it degraded over 95–99% of the total tetracycline in 24 h, indicating a much higher capability of resistance and degradation than those of the mixed culture. Therefore, we achieved the targeted degradation of tetracycline via a synthetic biology approach, and, as the tetracycline was successfully detoxified, our whole-cell device can be utilized as a pretreatment module coupled with traditional biological processes for the treatment of wastewater with high concentrations of tetracycline. Unlike tetracycline, to which only TetX has been reported to confer resistance by detoxification, aminoglycoside, amphenicol, and β-lactam antibiotics are mainly defended by enzymatic deactivation^37^. Therefore, our proposed whole-cell biodevice can be easily adopted and generalized for the degradation of various types of antibiotics.

Originated from model host, our designed biodevice can be easily cultivated, genetically manipulated and further engineered for additional functions. Moreover, this strategy can be generalized for other environmental benefits through general methodology, including codon-optimization, gene synthesis, and expression regulation using platform toolkits in model hosts. One of the concerns of synthetic routines, however, might be the introduction of heterologous and synthetic DNAs and the leaking risks of antibiotics resistance genes^23^. To this end, the CRISPR system provides a powerful tool to control the introduced gene programmably^28,38^, and we will discuss another prototypal strain with combined functional modules and a genetic regulation circuit soon.

## Methods

### Strains and media

*E. coli* DH5α (Takara Biotech, Dalian, China) was used for molecular cloning and maintaining the plasmids with antibiotic markers. *E. coli* BW25113 (CGSC#7636) was used as the model host to test whether our strategy worked. *E. coli* MG1655 (CGSC#6300) and *E. coli* BL21 (CGSC#12504) were employed for the detoxification assay. *S. oneidensis* MR-1 was a generous gift from Professor Han-Qing Yu (University of Science and Technology of China, Hefei, China). Luria-Bertani (LB) medium was used for all overnight cultures and most experiments unless otherwise noted. LB medium consisted of 10 g tryptone (Shangon Biotech, Shanghai, China), 5 g yeast extract (Shangon Biotech, Shanghai, China), and 10 g NaCl per liter of H_2_O. M9 medium contained 12.8 g/L of Na_2_HPO_4_, 3 g/L of KH_2_PO_4_, 0.5 g/L of NaCl, 1 g/L of NH_4_Cl, 0.24 g/L of MgSO_4_, 0.01 g/L of CaCl_2_ and 4 g/L glucose. To maintain plasmids with ampicillin resistance, 100 µg/mL of ampicillin was added to the media when necessary. *L*-arabinose was added to induce P_BAD_ promoters in both overnight culture and experimental cultures and to overcome the repression effects of glucose on P_BAD_ promoters via pre-accumulation of TetX, when presented in specific tests. Tetracycline (tetracycline-HCl, Aladdin Reagent, Shanghai, China) was added at different concentrations (0, 5, 10, 20 and 50 μg/mL) to evaluate the resistance and degradation capability of TetX. All the experiments were conducted in a biosafety level 1 (BSL-1) laboratory.

### Gene synthesis and plasmid construction

The *tetX* gene from *B. fragilis* was codon-optimized via JCat (http://www.jcat.de) for better expression in *E. coli*. Then, the *tetX.co* gene was synthesized (BGI, Beijing, China) and fused to the pBAD backbones with P_J23119_ and P_BAD_ promoters, generating pTc and pTi, respectively. The plasmids were constructed via Gibson assembly according to the manufacturer’s protocols (NEB, USA), and the transformation and selection of *E. coli* with designed plasmids were carried out following the standard protocols. The plasmids pcrRNA.con (Addgene 61285) and pcrRNA.ind (Addgene 61284) were used as the templates to provide backbones^39^. The sequence of *tetX.co* and all primers used in this study were summarized in Table SI.

### Cell growth tests in the presence of tetracycline

To evaluate the antimicrobial effects of tetracycline, cells were first cultivated in LB medium overnight at 37 °C and 200 rpm, and then were inoculated into fresh LB medium with different concentrations of tetracycline. The initial optical densities at 600 nm (OD_0_) and those at 12 h (OD_12_) were recorded with a UV-2000 spectrophotometer (UNICO, USA). The growth properties of *E. coli* were represented by OD_12_/OD_0_.

### Characterization of tetracycline conversion

The conversion of tetracycline was characterized via UV-Vis spectrophotometric scanning and mass spectrometric (MS) analysis. The BW25113 with pTc and pTi, respectively, and BW25113 with an empty plasmid were cultured overnight and inoculated into M9 medium with 50 μg/mL of tetracycline. Samples were taken at different time intervals and filtered for UV-Vis analysis. The UV-Vis scanning ranged from 240 nm to 480 nm and the absorbance was recorded every 0.5 nm. After 24 h, the final culture was harvested and filtered for MS analysis. The MS with a LCQ Fleet (Thermos, USA) was used, and ESI (electron spray ionization) was employed with a spray voltage set at 4,000 V. The sheath gas flow was 30 arb, aux gas flow was 10 arb, and the capillary temperature was 320 °C. The MS was obtained from 100 Da to 500 Da in the negative ion mode, and the results were shown in relative abundance of negative ions from 400 to 500 m/z (ratio of mass to charge). The concentrations of tetracycline were measured using HPLC (LC-20AT, Shimadzu, Japan) coupled with a reversed-phase ODS-C18 column and UV detector at the wavelength of 360 nm using a mixture of 0.01 mol/L oxalic acid solution/acetonitrile/methanol 80:16:4 (v/v/v) as mobile phase at a flow rate of 1 mL/min^33^.

### Detoxification assay via conditioned media (CM)

The detoxification capability of strains with *tetX.co* was analyzed using CM^40^. BW25113 and BW25113 with pTi were cultured overnight and inoculated into M9 and LB media with 50 μg/mL of tetracycline. After 24 h, the culture was harvested by centrifugation at 5,000 rpm and filtered through 0.22-μm cellulose acetate membranes. The filtered cultures were regarded as CM. To fully explore the detoxification functions of TetX, glucose (4 g/L) was added into the CM from LB culture as an additional carbon source to support cell growth. The results were represented by OD_12_/OD_0_ of tested strains.

## Electronic supplementary material

Supplementary Information
