# Supplementary material for: Synthetic Whole-Cell Biodevices for Targeted Degradation of Antibiotics
Source: Sci Rep. 2018 Feb 13;8:2906. doi: 10.1038/s41598-018-21350-9 (PMC5811551; doi:10.1038/s41598-018-21350-9)
Supplement: Supplementary file 1 — Supplementary Information [file 41598_2018_21350_MOESM1_ESM.pdf]

Supplementary Information

Synthetic Whole-Cell Biodevices for Targeted Degradation of Antibiotics

Peng-Fei Xia[a], Qian Li[a], Lin-Rui Tan[a], Miao-Miao Liu[b], Yong-Su Jin[c,d], Shu-Guang Wang[a,*]

[a] School of Environmental Science and Engineering, Shandong University. 27 Shanda Nanlu, Jinan, 250100, P.R. China

[b] Department of Civil and Environmental Engineering, University of Illinois at Urbana-Champaign, 205 North Mathews Ave. Urbana, IL 61801, United States

[c] Carl R. Woese Institute for Genomic Biology, University of Illinois at Urbana-Champaign, 1206 West Gregory Drive, Urbana, IL 61801, United States

[d] Department of Food Science and Human Nutrition, University of Illinois at Urbana-Champaign, 905k South Goodwin Avenue, Urbana, IL 61801, United States

Table SI Sequences of tetX.co and primers used in this study.

Name	Sequences
tetX.co	ATGACCATGCGTATCGACACCGACAAACAGATGAACCTGCTGTCTGACAAAAAC<br>GTTGCTATCATCGGTGGTGGTCCGGTTGGTCTGACCATGGCTAAACTGCTGCAGC<br>AGAACGGTATCGACGTTTCTGTTTACGAACGTGACAACGACCGTGAAGCTCGTA<br>TCTTCGGTGGTACTCTGGACCTGCACAAAGGTTCTGGTCAGGAAGCTATGAAAA<br>AAGCTGGTCTGCTGCAGACCTACTACGACCTGGCTCTGCCGATGGGTGTTAACA<br>TCGCTGACAAAAAAGGTAACATCCTGTCTACCAAAAACGTTAAACCGGAAAAC<br>CGTTTCGACAACCCGGAAATCAACCGTAACGACCTGCGTGCTATCCTGCTGAAC<br>TCTCTGGAAAACGACACCGTTATCTGGGACCGTAAACTGGTTATGCTGGAACCG<br>GGTAAAAAAAAATGGACCCTGACCTTCGAAAACAAACCGTCTGAAACCGCTGA<br>CCTGGTTATCCTGGCTAACGGTGGTATGTCTAAAGTTCGTAAATTCGTTACCGAC<br>ACCGAAGTTGAAGAAACCGGTACTTTCAACATCCAGGCTGACATCCACCAGCCG<br>GAAATCAACTGCCCAGGTTTCTTCCAGCTGTGCAACGGTAACCGTCTGATGGCT<br>TCTCACCAGGGTAACCTGCTGTTCGCTAACCCGAACAACAACGGTGCTCTGCAC<br>TTCGGTATCTCTTTCAAAACCCCGGACGAATGGAAAAACCAGACCCAGGTTGAC<br>TTCCAGAACCGTAACTCTGTTGTTGACTTCCTGCTGAAAGAATTTTCTGACTGGG<br>ACGAACGTTACAAAGAACTGATACACACCACCCTGTCTTTCGTTGGTCTGGCTA<br>CCCGTATCTTCCCGCTGGAAAAACCGTGGAAATCTAAACGTCCGCTGCCGATAA<br>CCATGATAGGTGACGCTGCTCACCTGATGCCGCCGTTCGCTGGTCAGGGTGTTA<br>ACTCTGGTCTGGTTGACGCTCTGATACTGTCTGACAACCTGGCTGACGGTAAATT<br>CAACTCTATCGAAGAAGCTGTTAAAAACTACGAACAGCAGATGTTCATGTACGG<br>TAAAGAAGCTCAGGAAGAATCTACCCAGAACGAAATCGAAATGTTCAAACCGG
Pc1-tetX	GCTAGCGAATTCGAGCTCGGTACCACCTCGATGACCATGCGTATCGACACCGAC<br>AAACAG
Pc2-tetX	AATTCTGTTTTATCAGACCGCTTCTGCGTTTTAAACGTTCAGCAGCTGCTGGAAG<br>GTGAA
Pc1-p	CTGTTTGTCGGTGTCGATACGCATGGTCATCGAGGTGGTACCGAGCTCGAATTCG<br>CTAGC
Pc2-p	TTCACCTTCCAGCAGCTGCTGAACGTTTAAAACGCAGAAGCGGTCTGATAAAAC<br>AGAATT
Pi1-tetX	ATACCCGTTTTTTTGGGCTAGCGAATTCGAATGACCATGCGTATCGACACCGACA<br>AACAG
Pi2-tetX	AATTCTGTTTTATCAGACCGCTTCTGCGTTTTAAACGTTCAGCAGCTGCTGGAAG<br>GTGAA
Pi1-p	CTGTTTGTCGGTGTCGATACGCATGGTCATTCGAATTCGCTAGCCCAAAAAAACG<br>GGTAT
Pi2-p	TTCACCTTCCAGCAGCTGCTGAACGTTTAAAACGCAGAAGCGGTCTGATAAAAC<br>AGAATT
